# CryoEM at **IUCrJ**: a new era

**DOI:** 10.1107/S2052252515023738

**Published:** 2016-01-01

**Authors:** Sriram Subramaniam, Werner Kühlbrandt, Richard Henderson

**Affiliations:** a Laboratory of Cell Biology, Center for Cancer Research, National Cancer Institute, National Institutes of Health, Bethesda, MD 20892, USA; b Department of Structural Biology, Max Planck Institute of Biophysics, Frankfurt, 60538, Germany; c MRC Laboratory of Molecular Biology, Francis Crick Avenue, Cambridge, CB2 0QH, UK

**Keywords:** electron cryomicroscopy, electron tomography, single particle, cryoEM, overview

## Abstract

In this overview, we briefly outline recent advances in electron cryomicroscopy (cryoEM) and explain why the journal **IUCrJ**, published by the International Union of Crystallography, could provide a natural home for publications covering many present and future developments in the cryoEM field.

## Abstract 2

In this overview, we briefly outline recent advances in electron cryomicroscopy (cryoEM) and explain why the journal **IUCrJ** can provide a natural home for publications covering many present and future developments in the cryoEM field.

## 1. Overview and relationship of cryoEM to **IUCrJ**   

The International Union of Crystallography (IUCr) was founded in 1947. It provided a worldwide forum to bring together the interests of a wide range of scientists using X-ray crystallography and diffraction to study structures in fields ranging from chemistry and mineralogy to biology, physics and materials science. It has been enormously successful and has managed to remain a grass-roots organization with strong community support, an open-access structure and triennial Congresses. The names of the first presidents are legendary – Bragg, Bijvoet, Wyckoff, Wyart, Ewald, Bernal, Lonsdale, Belov, Guinier, Hodgkin. Its first journal, *Acta Crystallographica* was launched in 1948. The portfolio of IUCr publications gradually expanded to include *Acta Cryst.* *A*, *B*, *C*, *D*, *E*, *F*, *Journal of Applied Crystallography*, *Journal of Synchrotron Radiation* and the *International Tables*. The range of research interests within IUCr also expanded to encompass electron and neutron diffraction, and now, with the encouragement of its editorial board, cryoEM.

**IUCrJ** is the most recent journal in the IUCr portfolio, having started only in January 2014 with its first edition, to mark the International Year of Crystallography (IYCr2014) and the 100th anniversary of the award of the first Nobel Prize related to crystallography to Max von Laue. The inaugural editorial (Hasnain, 2014 ▸) set out the range of topics to be covered by the journal. The topics included biology, chemistry, crystal engineering, materials, physics and a wide range of scientific, methodological and technical approaches, with coverage of synchrotron and neutron sources as well as physics and free electron lasers (FELs). Electron cryomicroscopy (cryoEM) was not mentioned when **IUCrJ** was launched, perhaps because it might have been difficult to predict the rapid pace of major advances in the cryoEM field even two years ago. It is therefore entirely appropriate that **IUCrJ** extends its reach to include cryoEM, and this was announced recently (Hasnain, 2015 ▸).

Traditionally, the journals of choice for publication of results from technical and biological aspects of cryoEM studies have included *Ultramicroscopy*, *Journal of Structural Biology*, *Structure*, *Journal of Molecular Biology*, *Proceedings of the National Academy of Sciences of the USA* and *EMBO Journal*, and more recently a huge increase in publications in journals that reach a broad biological audience such as *Nature*, *Science*, *Cell* and *eLife*. CryoEM studies of course have formed only a small part of the scope of most of the journals. We expect that **IUCrJ** will similarly develop its own particular emphasis in the cryoEM area and we enthusiastically encourage potential authors to submit manuscripts in which cryoEM results, techniques and methodological advances are the main fare, while being mindful of the breadth of **IUCrJ** readership.

## 2. Recent advances in cryoEM   

**Figure 1:**
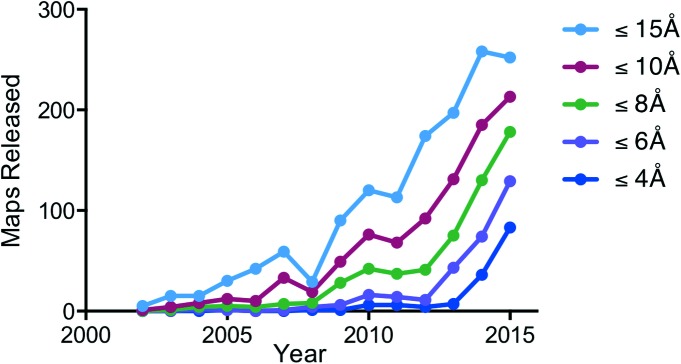
Growth in number of cryoEM structures deposited at the Electron Microscopy Data Bank since 2002 (taken from the PDB website: http://pdbe.org/emstats). There is exponential growth at all resolution ranges, and a dramatic increase in the number of structures with resolutions better than 4 Å. Earlier studies that represent landmarks in the development of single-particle cryoEM were the hepatitis B capsid determined at 7.4 Å resolution (Böttcher *et al.*, 1997 ▸; Conway *et al.*, 1997 ▸) and the *E.coli* 70S ribosome at 11.5 Å resolution (Gabashvili *et al.*, 2000 ▸).

Since 2012, the advent of new electron detectors and improved computational programs together with substantial improvements in the electron microscopes themselves has produced an avalanche of new structures (Kühlbrandt, 2014 ▸), publications and coordinate depositions in the Electron Microscopy Data Bank (EMDB) and the Protein Data Bank (PDB), as summarized in Fig. 1 ▸. The productivity and power of the method has attracted many newcomers, from cell biologists who have a more biological emphasis, to those from adjacent disciplines such as crystallography and NMR spectroscopy who already have extensive depth of experience in structural biology. The recent successes span a wide range and include small proteins (<500 kDa), medium- and large-sized complexes (>1 MDa) as well as flexible and multi-domain protein complexes, several of which have proved to be resistant to analysis by X-ray crystallography over the years. Further, the level of automation in all aspects of the workflow has increased, making it easier for new users to adopt the method and to use it successfully.

**Figure 2:**
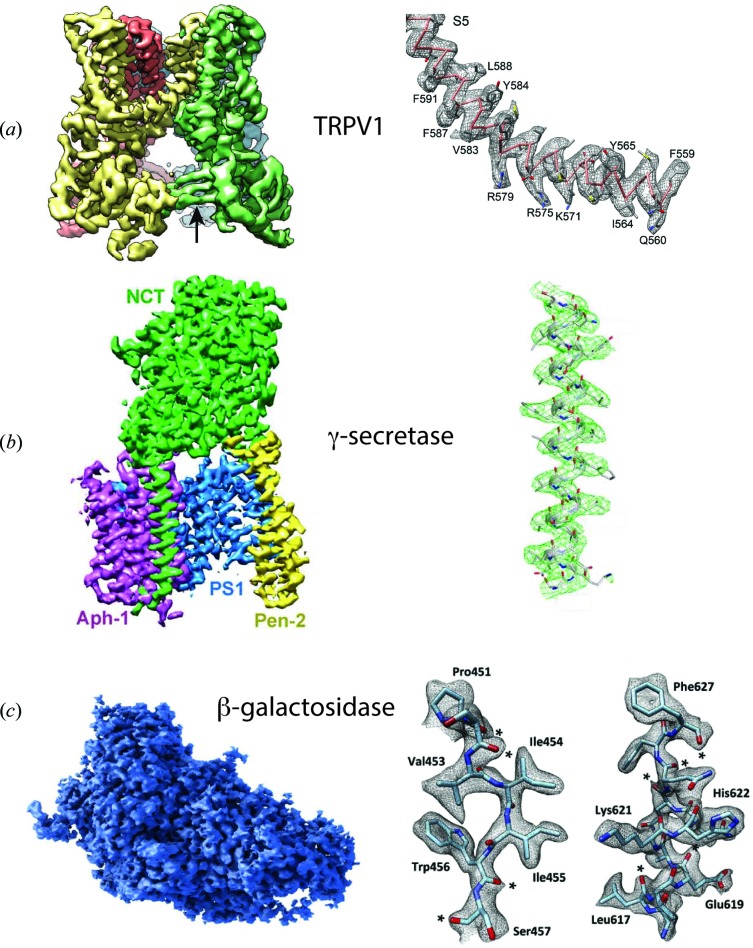
Selected examples of structures with sizes below 500 kDa. (*a*) TRPV1 temperature-sensing channel also called the capsaicin receptor (Liao *et al.*, 2013 ▸) at 3.4 Å resolution, (*b*) γ-secretase at 3.4 Å resolution (Bai *et al.*, 2015 ▸) and (*c*) β-galactosidase at 2.2 Å resolution (Bartesaghi *et al.*, 2015 ▸). Not on same scale.

**Figure 3:**
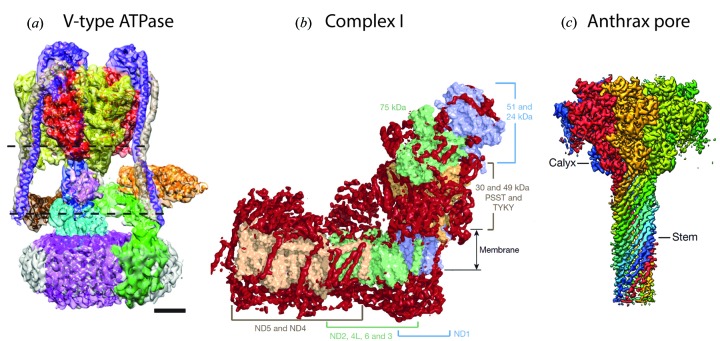
Examples of selected medium-sized membrane protein structures. (*a*) Yeast V-type ATPase at 7 Å resolution (Zhao *et al.*, 2015 ▸), (*b*) mitochondrial Complex I (Vinothkumar *et al.*, 2014 ▸) at 5 Å resolution, and (*c*) the anthrax protective antigen pore at 2.9 Å resolution (Jiang *et al.*, 2015 ▸). Scale bar is 25 Å.

**Figure 4:**
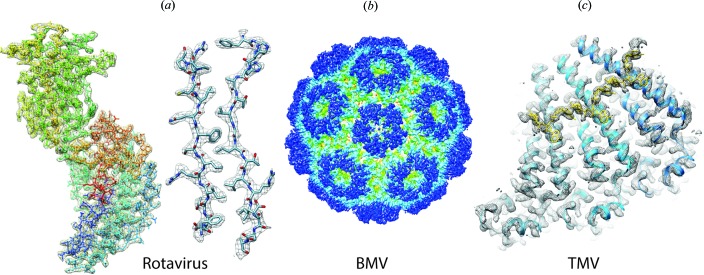
Examples of some large structures. (*a*) Human rotavirus (Grant & Grigorieff, 2015 ▸), (*b*) brome mosaic virus (Wang *et al.*, 2014 ▸) and (*c*) TMV (Fromm *et al.*, 2015 ▸), at resolutions of 2.6 Å, 3.8 Å and 3.4 Å, respectively. Not on same scale.

Representative examples of structures of small proteins resolved at high resolution include the membrane proteins TRPV1 temperature-sensing channel (Liao *et al.*, 2013 ▸) and γ-secretase (Bai *et al.*, 2015 ▸), and the enzyme β-galactosidase (Bartesaghi *et al.*, 2015 ▸) (Fig. 2 ▸). Examples of medium-sized membrane protein structures, where previously only poorly ordered crystals had been obtained, or where conformational heterogeneity was a problem, are yeast V-type ATPase (Zhao *et al.*, 2015 ▸), mitochondrial Complex I (Vinothkumar *et al.*, 2014 ▸) and anthrax protective antigen pore (Jiang *et al.*, 2015 ▸) (Fig. 3 ▸). Examples of some large structures, including icosahedral viruses where the resolution has been greatly improved in conjunction with the use of smaller-sized data sets compared to earlier analyses include human rotavirus (Grant & Grigorieff, 2015 ▸), brome mosaic virus (Wang *et al.*, 2014 ▸) and TMV (Fromm *et al.*, 2015 ▸) (Fig. 4 ▸). In addition, great progress has been made in analysis of helically ordered assemblies as shown by reports of numerous structures at resolutions close to 3 Å (Egelman, 2015 ▸). It is also worth noting that, while many single-particle assemblies can in principle be crystallized, helical structures including helical viruses have symmetry constraints that are incompatible with crystallization.

A brief overview of advances in cryoEM would not be complete without mentioning the achievements and potential of electron cryotomography (cryoET). The application of cryoET to understand the macromolecular architectures of eukaryotic (Medalia *et al.*, 2002 ▸) and prokaryotic (Gan & Jensen, 2012 ▸) cells has shown that this method has enormous potential for investigating structures at the sub-cellular level. It is also possible to carry out sub-tomogram averaging in three dimensions to improve the resolution of structure determination of structures that are found in multiple copies in each tomogram, such as the work on the structure of HIV Env trimers on the surface of infectious HIV particles (Liu *et al.*, 2008 ▸). In principle, the averaging of sub-tomogram volumes should eventually produce maps at resolutions comparable to those produced using single-particle cryoEM methods.

## 3. What’s in the pipeline?   

During the next few years, we expect that technical advances will make cryoEM more powerful and versatile than it is at present. We anticipate that further advances will occur in detector technology, phase plates, *Cc* correctors, computing power and algorithms, design of better specimen supports, and improved imaging strategies, although there are unsolved problems in each of these areas that might take a few years to overcome. We briefly discuss each of these six areas below.

The three companies that produce the currently available direct electron detectors commercially are Gatan, FEI and Direct Electron. In each case, their detectors produce images using 300 keV electrons that are significantly better than obtained with film (McMullan *et al.*, 2014 ▸). They are all based on the same CMOS/MAPS technology in which each frame of the exposure is read out in rolling-shutter mode as a ‘movie’. The DQE at half the Nyquist frequency is in the range 40–60% but typically drops to ~25% at Nyquist. Higher DQE values than these can only be obtained by operating in counting mode where the image of each incident electron is substituted by an idealized single count, so all future improved detectors will need to operate in counting mode. High frame rates are required for counting, to avoid double hits on individual pixels or very long exposures times, and at present only the Gatan K2, when operated in counting mode, can produce a DQE(0) as high as 80% in conjunction with reasonably small exposure times. The K2 detector frame rate is about 10 times higher than that available with the two other detector brands. If the arrival point of each incident electron can be determined to a sub-pixel accuracy with sufficient precision, there is no reason why the DQE(ω) should not approach 100% without much drop at Nyquist, and the detectors might then be usable beyond Nyquist in super-resolution mode. The K2 detector already allows operation in super-resolution mode, but an increased DQE at and beyond Nyquist should make it and other detectors that can operate in the counting mode much more effective.

Microscopes capable of using phase plates are available from both FEI and JEOL, and several groups are working to improve a number of different approaches to phase plate design although their use still requires a high degree of specialized expertise (Danev & Nagayama, 2008 ▸; Glaeser *et al.*, 2013 ▸; Walter *et al.*, 2015 ▸). Also, given that bright-field defocus phase contrast can produce images that transmit full contrast at CTF maxima, the advantage of the phase plate is to provide increased contrast where the bright-field CTF has zeroes, including at very low spatial frequency. The effect of a phase plate should be to provide up to a twofold increase in average signal-to-noise ratio and this needs be done with minimal losses due to inelastic scatter by the phase plate material. For example, the use of the Volta phase plate from FEI that is based on a continuous carbon film involves some losses due to electron scattering by the carbon film. Despite these challenges, the realisation of a robust and practical phase plate has great promise and remains an active research area. Current cryoEM work with phase plates has already achieved sub-nanometre resolution and there is reason to hope the new phase plate designs will enable near-atomic resolution in the near future.

Chromatic aberration (Cc) correctors offer the opportunity to enhance the image contrast of the elastic image by ensuring that the elastically and inelastically scattered electrons are focused in the same way (Kabius *et al.*, 2009 ▸), thus increasing rather than decreasing the signal. Inelastically scattered electrons normally add a more-or-less homogeneous background fog to the image, which reduces the signal-to-noise ratio. The degree of improvement to be expected depends on the proportion of inelastic scattering. If this is small, for example with a specimen consisting of a thin layer of amorphous ice, then the improvement will be minimal. Conversely, if the ice layer is too thick, the image will be limited by multiple elastic scattering and will contain little high-resolution information. Thus the main advantage of a Cc corrector is likely to be for specimens with ice thicknesses in the range of 1500–3000 Å, although the cost of adding this accessory to a microscope is currently very high.

Although many years of development have produced programs with sophisticated image processing algorithms, we should not rule out further improvements, for example from more powerful image classification, that take advantage of the steady increase in computing power. Efforts to port existing image processing programs such as *FREALIGN* and *RELION*, that were developed in the context of using central processor units (CPUs), to use graphical processor units (GPUs) are already underway or available (Li *et al.*, 2010 ▸). Since several thousand GPUs are available commercially on video processor units, this could greatly increase the speed of single-particle cryoEM image processing and decrease the cost of computation.

There have been substantial advances in methods to make stable supports that move much less during electron irradiation. For example Russo & Passmore (2014 ▸) have shown that holey gold films move about 50× less during electron irradiation than the engineered holey carbon films that most researchers in the cryoEM field use (Ermantraut *et al.*, 1997 ▸). We expect that there will be increasing availability of stable supports with a wide range of hole sizes, which should reduce the extent of beam-induced specimen motion.

Finally, large gains might be made if the problem of beam-induced specimen motion (with its accompanying image blurring) could be overcome. All recently published cryoEM structure determinations have reported that the quality of the first few frames of a movie series recorded with a direct electron detector is poorer than those frames in the window between 3–4 e Å^−2^ and 10–15 e Å^−2^ (*e.g.* Scheres, 2014 ▸; Bartesaghi *et al.*, 2015 ▸). At electron doses above 15 e Å^−2^, radiation damage causes a decline in the information content, as expected. However, electron diffraction from thin crystals of organic or biological specimens shows that the first 3–4 e Å^−2^ should represent the very best part of the exposure in terms of recovering structural information (Stark *et al.*, 1996 ▸; Fujiyoshi, 1998 ▸). A solution, or even a consensus on the cause of the contrast loss in the first few frames has not yet been reached, except to say that some combination of beam-induced physical motion and beam-induced specimen charge build-up must be responsible. A solution such as spot-scan imaging (Bullough & Henderson, 1987 ▸; Downing, 1991 ▸), possibly accompanied by paraxial charge compensation (Berriman & Rosenthal, 2012 ▸), may help to solve the problem. A reduction in this image blurring during the first few frames by even a factor of two or three would produce a substantial reduction in the number of images needed to reach high resolution.

## 4. Long-term dreams   

If all the known technical problems that remain to be solved in the cryoEM area are overcome, then the approach should yield results that match up to theoretical expectations (Henderson, 1995 ▸). It should be possible to determine high-resolution structures of protein complexes small and large, from images of only a few thousand particles or less, and to resolve multiple conformational states at high resolution. It will be particularly satisfying if the structure of human haemoglobin (64 kDa MW), whose structure determination by Max Perutz launched the last five decades of protein crystallography could be determined at 3 Å resolution by single-particle cryoEM. Another challenge that might be met could be the analysis of flexible, multi-domain protein structures by an iterative approach where the architecture is determined by solving structures of domains progressively. Thus, the largest domain could be tackled first, then ‘subtracted’ computationally from the experimental image to get at the next largest domain, and to repeat this process sequentially until the complete structure as well as overall architecture has been determined.

We hope that **IUCrJ** will be a key journal that can ride the wave of all the expected (and unexpected) technical advances that we believe will continue to make cryoEM methods even more powerful in the coming decade. The journal can act as a vehicle to publicize these advances and help the cryoEM field to move forward coherently. CryoEM itself may become the first choice method at the start of any structural biology project, since it requires a smaller quantity of material that is less pure, less stable and less homogeneous than needed for many other methods. It may even become the dominant method in structural biology in the future.
